# Management of traumatic meniscus tears: the 2019 ESSKA meniscus consensus

**DOI:** 10.1007/s00167-020-05847-3

**Published:** 2020-02-13

**Authors:** Sebastian Kopf, Philippe Beaufils, Michael T. Hirschmann, Niccolò Rotigliano, Matthieu Ollivier, Helder Pereira, Rene Verdonk, Nikica Darabos, Panagiotis Ntagiopoulos, David Dejour, Romain Seil, Roland Becker

**Affiliations:** 1 Center of Orthopaedics and Traumatology, Brandenburg Medical School Theodor Fontane, Hochstr. 29, 14770 Brandenburg an der Havel, Germany; 2 Orthopaedics Department, Centre Hospitalier de Versailles, Le Chesnay, France; 3 Department of Orthopaedic Surgery and Traumatology, Kantonsspital Baselland (Bruderholz, Liestal, Laufen) and University of Basel, Basel, Switzerland; 4 Department of Orthopedics and Traumatology, Institute of Movement and Locomotion, St. Marguerite Hospital, 270 Boulevard Sainte Marguerite, BP 29, 13274 Marseille, France; 5 Orthopedic Department Centro Hospitalar Póvoa de Varzim, Vila do Conde and ICVS/3 Bs Associated Laboratory, Minho University, Braga, Portugal; 6 Department of Orthopaedic Surgery and Traumatology, University Hospital Erasmus Bruxelles, Bruxelles, Belgium; 7 Department of Traumatology, Bone and Joint Surgery, Clinic of Surgery, University Hospital Centre Zagreb, Zagreb, Croatia; 8 Hip and Knee Unit, Mediterraneo Hospital, 10 Ilias Street, 16675 Glyfada, Athens Greece; 9 Orthopaedic Department, Lyon-Ortho-Clinic, Clinique de La Sauvegarde, Avenue Ben Gourion, 69009 Lyon, France; 10 Service de Chirurgie Orthopédique, Centre Hospitalier de Luxembourg-Clinique d’ Eich, 78, 1460 Rue d’ Eich, Luxembourg; 11 Luxembourg Institute of Health, 78, 1460 Rue d’Eich, Luxembourg; 12 Department of Orthopedics and Traumatology, Centre of Joint Replacement, Hospital Brandenburg, Medical School “Theodor Fontane”, Hochstrasse 29, 14770 Brandenburg/Havel, Germany

**Keywords:** Meniscus, Traumatic tear, Repair, Management, Consensus, Meniscus preservation

## Abstract

**Purpose:**

The importance of meniscus integrity in the prevention of early osteoarthritis is well known, and preservation is accepted as the primary goal. The purpose of the ESSKA (European Society for Sports Traumatology, Knee Surgery and Arthroscopy) European consensus on traumatic meniscus tears was to provide recommendations for the treatment of meniscus tears based on both scientific evidence and the clinical experience of knee experts.

**Methods:**

Three groups of surgeons and scientists elaborated and ratified the so-called formal consensus process to define the recommendations for the management of traumatic meniscus tears. A traumatic meniscus tear was defined as a tear with an acute onset of symptoms caused by a sufficient trauma. The expert groups included a steering group of eight European surgeons and scientists, a rating group of another nineteen European surgeons, and a peer review group. The steering group prepared twenty-seven question and answer sets based on the scientific literature. The quality of the answers received grades of A (a high level of scientific support), B (scientific presumption), C (a low level of scientific support) or D (expert opinion). These question and answer sets were then submitted to and evaluated by the rating group. All answers were scored from 1 (= totally inappropriate) to 9 (= totally appropriate) points. Thereafter, the comments of the members of the rating group were incorporated by the steering group and the consensus was submitted to the rating group a second time. Once a general consensus was reached between the steering and rating groups, the finalized question and answer sets were submitted for final review by the peer review group composed of representatives of the ESSKA-affiliated national societies. Eighteen representatives replied.

**Results:**

The review of the literature revealed a rather low scientific quality of studies examining the treatment of traumatic meniscus tears. Of the 27 questions, only one question received a grade of A (a high level of scientific support), and another received a grade of B (scientific presumption). The remaining questions received grades of C and D. The mean rating of all questions by the rating group was 8.2 (95% confidence interval 8.1–8.4). A general agreement that MRI should be performed on a systematic basis was not achieved. However, MRI was recommended when arthroscopy would be considered to identify concomitant pathologies. In this case, the indication for MRI should be determined by a musculoskeletal specialist. Based on our data, stable left in situ lateral meniscus tears appear to show a better prognosis than medial tears. When repair is required, surgery should be performed as early as possible. Evidence that biological enhancement such as needling or the application of platelet-rich plasma would improve healing was not identified. Preservation of the meniscus should be considered as the first line of treatment because of an inferior clinical and radiological long-term outcome after partial meniscectomy compared to meniscus repair.

**Discussion:**

The consensus was generated to present the best possible recommendations for the treatment of traumatic meniscus tears and provides some groundwork for a clinical decision-making process regarding the treatment of meniscus tears. Preservation of the meniscus should be the first line of treatment when possible, because the clinical and radiological long-term outcomes are worse after partial meniscectomy than after meniscus preservation. The consensus clearly states that numerous meniscus tears that were considered irreparable should be repaired, e.g., older tears, tears in obese patients, long tears, etc.

**Level of evidence:**

II

## Introduction

The nomenclature of traumatic and degenerative chronic meniscus injuries should be distinguished based on their etiology. The ESSKA European meniscus consensus group defined traumatic meniscus injury as a ‘meniscus tear’, which is associated with a sufficient knee injury and a sudden onset of knee pain, whereas a ‘meniscus lesion’ is a degenerative meniscus tear marked by a slow progression of tissue degeneration without a history of an acute trauma [1]. The main types of meniscus tears are vertical tears, such as longitudinal (including bucket handle) and radial tears [2] (including flap), and posterolateral root tears. Although debate exists regarding whether tears of the meniscus ramp should also be included under the purview of traumatic meniscus tears, they are generally accepted to occur at a ligamentous connection between the posterior horn of the medial meniscus and the tibial plateau. These meniscal ramp tears often do not affect the actual meniscus tissue and thus were not counted as true meniscus tears in this consensus. In contrast, horizontal lesions are not considered traumatic meniscus tears because of their rather degenerative nature, even if they occur in younger patients [3–5]. These lesions are caused by repetitive microtrauma and degeneration of the tissue in conjunction with or without osteoarthritis (OA). The two pathologies, traumatic and degenerative, must be distinguished because of the fundamental differences in optimal management.

Traumatic meniscus tears may occur in isolation but are commonly detected in conjunction with ligament injuries. The odds ratios of developing OA after isolated meniscus injuries or combined injuries of both the anterior cruciate ligament (ACL) and the meniscus are approximately 6.3 and 6.4, respectively [6]. The main surgical treatment options are arthroscopic partial meniscectomy or meniscus repair. The latter led to less OA, a higher level of activity and higher patient satisfaction in the long term in some studies [7–10]. Despite this evidence, a significant mismatch has been identified between the incidence of repairable meniscus tears and the repair rates in clinical practice. The incidence of traumatic meniscus tears in patients with a torn ACL ranged between 57 and 80% [11, 12]. Although more than 30% of meniscus tears are estimated to be suitable for repair, less than 10% are repaired [2, 13]. For instance, in France, 1,564,461 meniscectomies and 63,142 meniscus repairs were performed between 2005 and 2017, resulting in a repair rate of only 4% [14]. Nevertheless, repair rates appear to increase over time. In the US, an analysis by the database of the American Board of Orthopedic Surgery revealed that the percentage of meniscus repairs has increased by 37% per surgeon from 2004 to 2012, whereas the rate of meniscectomies decreased by 17% per surgeon over the same period [15].

In addition to OA, the time to return to activity or failure rates may affect the treatment decision. After partial, subtotal or even total meniscectomy, patients generally return to their normal daily activities within 2–4 weeks. In contrast, patients require significantly more time for recovery after meniscus repair. However, meniscus repairs have a greater potential for helping patients return to the same level of activity. Another frequently mentioned drawback of meniscal repairs is their higher risk of failure and thus early revision arthroscopy in the short (16.5 vs. 1.4%) and long term (20.7 vs. 3.9%) [8], both in adults and children or adolescents (repair: 18% vs. meniscectomy: 7%) [16]. Nevertheless, the clinical success rate of meniscus repairs substantially outweighs the failures, by 85%, as shown in a recent analysis of the literature [17].

The types of meniscus tears that are suitable for repair must be identified to perform the repair and avoid unnecessary partial meniscus resections to decrease the risk of OA and allow patients return to full activity. In our opinion, the contrast of high rates of successful meniscus repairs and its superiority to partial meniscectomy regarding OA development and the return to the preoperative level of activity and the low number of performed repairs compared to meniscectomies indicates an apparent lack of understanding about the importance of meniscus repair.

Therefore, this European consensus may help in the decision-making process for the treatment of patients with traumatic meniscus tears by also considering factors such as a high BMI, age of the meniscus tears and patients and biological treatment options. Thus, this consensus will help the surgeon to improve his decision-making process and subsequently improve patient outcomes.

## Materials and methods

A European meniscus consensus project was established by the European Society of Sports Traumatology, Knee Surgery and Arthroscopy (ESSKA) between 2014 and 2018, focusing on the management of traumatic meniscus tears. A traumatic meniscus tear was defined as a tear with an acute onset of symptoms caused by a sufficient trauma.

**Fig. 1:**
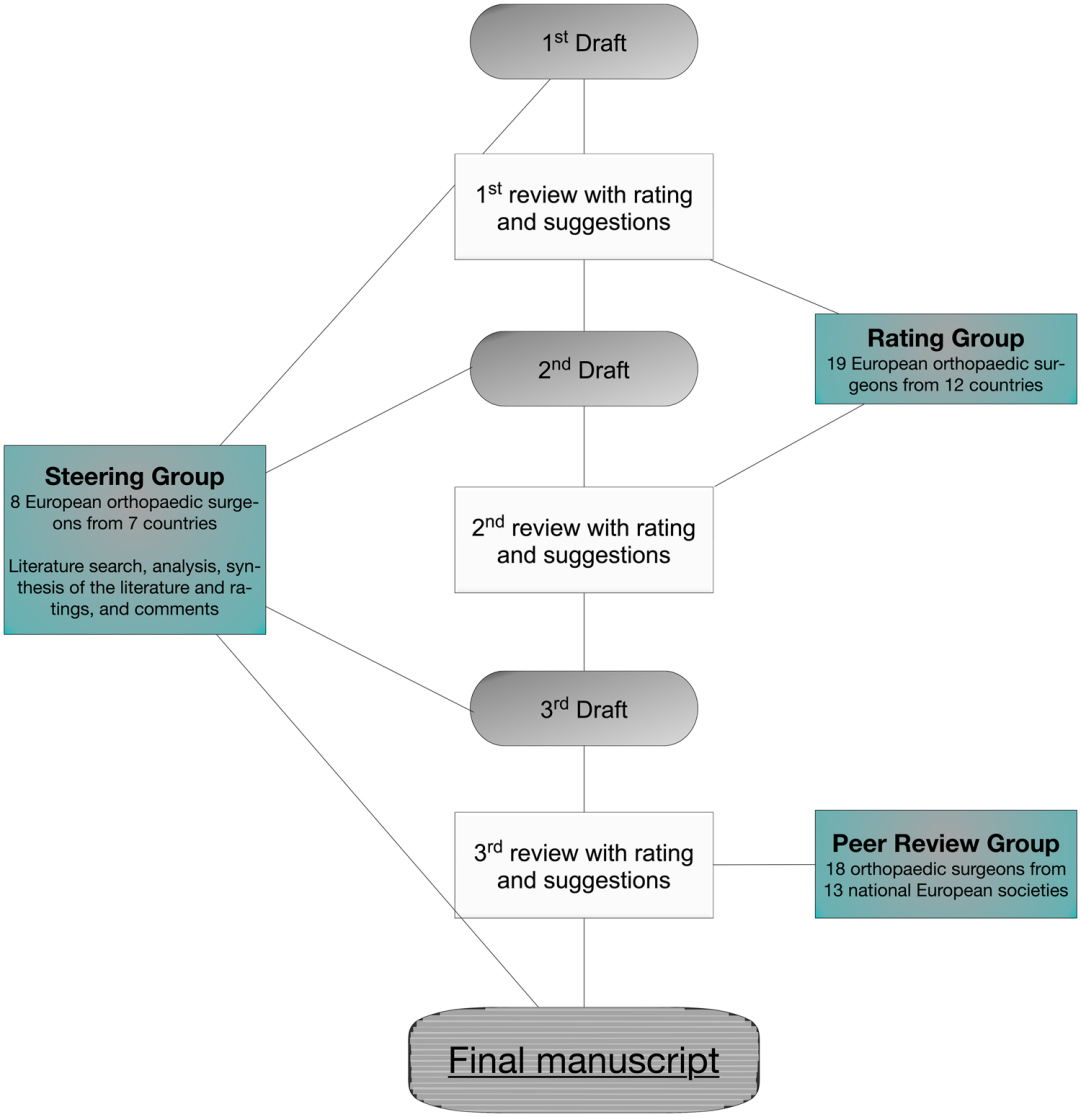
Flowchart of the procedure used to determine the ESSKA consensus for traumatic meniscus tears

The process of this consensus project was similar to the previously published ESSKA European degenerative meniscus consensus project (Fig. 1) [1]. Three groups of 45 experienced orthopedic surgeons and scientists (steering group, *n* = 8; rating group, *n* = 19; and peer review group, *n* = 18) were involved in the consensus process. First, a steering group was established that included eight expert knee surgeons under the leadership of three specialists with a special interest in the management of meniscus pathologies (PB, RB, and SK). The steering group was responsible for developing the questions and answers based on an extensive review of the literature using PubMed, Embase and Cochrane Central Register of Controlled Trials. The following terms were included in the search and used in different combinations: [meniscus], [tear], [injury], [trauma], [incidence], [lesion], [location], [diagnostic], [MRI], [treatment], [management], [arthroscopy], [repair], [suture], [debridement], [anterior cruciate ligament], [demographics], [healing], [second look] and [conservative management]. Additionally, references in the identified studies were searched for relevant studies. Clinical studies with levels of evidence ranging from one to five were included in this analysis. Animal and cadaver studies were excluded. Twenty-seven questions were developed according to four sections: 1. definition, 2. epidemiology, 3. diagnosis and 4. treatment. All questions were answered by the steering group based on the scientific literature. The quality of the answers was graded based on the quality of the available studies and was sorted into the appropriate grade of recommendation [18]. Grade A was defined as a high level of scientific support, grade B as a scientific presumption, grade C as a low level of scientific support, and grade D as an expert opinion. All question and answer sets were discussed and a consensus was achieved by the steering group according to the scientific grading. After a general agreement was achieved in the steering group, the questions were submitted to the rating group, which consisted of 19 European orthopedic surgeons, physiotherapists and scientists specialized in knee pathologies. Each member of the rating group was asked to score the question and answer sets according to the scientific evidence and their clinical experience on a Likert scale ranging from 1 (totally inappropriate) to 9 (totally appropriate) points. Suggestions from the participants were included after the first round. A revised draft was prepared and resubmitted to the rating group for final scoring. After the second draft was approved by both the steering and rating groups, the question and answer sets were sent to the national societies affiliated with ESSKA (*n* = 24). Eighteen surgeons from thirteen societies replied. The purpose of this unbiased peer review group was to evaluate the questions and answers of the manuscript after grading by the rating group to determine the feasibility, accessibility and readability of the proposed recommendations.

## Results

Answers were rated with an average of 8.2 points (95% confidence interval 8.1–8.4). The mean points assigned by each rater ranged from 6.9 to 8.8 points. Only 1 of the 27 answers received a grade of less than 7 points (exactly 6.9), and 5 answers received scores ranging from 7 to 7.9 points.

The majority of answers, including several questions with more than one graded answer, were evaluated as grade C (*n* = 27) or D (*n* = 10), indicating that a low level of scientific evidence is available for most of the answers. Only two answers were graded better than C, one with a grade of A and another with a grade of B. Two answers were not rated because of missing literature at the time the answers of this consensus were prepared.

## Questions and answers

### 1. Definition

#### 1. What is the definition of a traumatic meniscus tear?

A traumatic meniscus tear is a meniscus tear that is associated with a sufficient knee injury and a sudden onset of knee pain. Vertical tears, such as longitudinal (including bucket handle tears) and radial tears, are primarily included in this group [2]. Flap tears and mainly posterolateral root tears are also included. Tears of the meniscus ramp are also traumatic tears, but some debate persists regarding the definition. In general, they are believed to occur at a ligamentous connection between the posterior meniscus horns and the tibial plateau. They often do not affect the actual meniscus tissue and are thus not counted as true meniscus tears in this consensus. In general, horizontal lesions are not traumatic meniscus tears because of their rather degenerative nature (even in younger patients) [3-5] *(Grade D).*

#### 2. What is the definition of stable and unstable traumatic meniscus tears?

In unstable meniscus tears, the central part of the torn meniscus can be dislocated towards the joint space to the center (horizon) of the femoral condyle, thus evoking locking and sudden pain [19, 20]. The unstable meniscus fragment engages or is able to engage between the tibia plateau and the MCL or into the notch, or it is displaceable to at least approximately 5 mm [21]. A typical example is a longitudinal tear, which might temporarily evolve into a bucket handle tear. Another example might be a flap tear that engages between the femoral condyle and the tibial plateau [22–24]. In terms of partial or very short meniscus tears, a stable tear is defined as a tear that is not displaceable with the probe [25]. Radial tears are generally defined as unstable [24] *(Grade D).*

#### 3. What is the definition of a stable and unstable knee?

Functional instability is a symptom. Laxity is a measurable sign. In the current study, a stable knee has intact ligaments. This definition also includes a stabilized knee, e.g., after ACL reconstruction, although the success of the reconstruction was not fully considered in most of the studies [26, 27] *(Grade D).*

#### 4. Which classification should be used to describe the location of a meniscus tear?

**Fig. 2:**
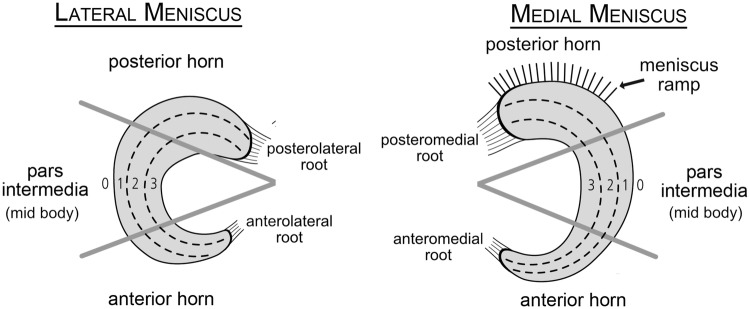
Newly proposed classification for the localization of meniscus tears (modified scheme, originally introduced by Cooper et al. and modified by Beaufils et al. [29, 69])

The meniscus should be classified into circumferential and radial zones (Fig. 2) [28, 29]. The radial zones have also been divided according to the vascularity in red–red, red–white, and white–white zones; however, this classification should be avoided, because vascularity changes throughout life and is often not directly assessable during surgery [30]. Furthermore, dividing the width of the meniscus into zones 0–3, as shown in Fig. 2, is a more objective and measurable approach *(Grade C).*

### Epidemiology

#### 5. What is the incidence of traumatic meniscus tears in stable knees?

For the general population, approximately 6% of acutely injured knees were reported to sustain a meniscus tear [31, 32]. The medial meniscus is involved in 75% of these cases. Numbers of acute meniscus injuries per 1000 inhabitants per year range from 0.5 to 0.7 [32, 33]. Men (0.7/1000 inhabitants/year) are more frequently affected than women (0.3/1000 inhabitants/year) [33]. Approximately 15% of athletes with acute knee trauma and hemarthrosis sustain isolated meniscus tears with a higher ratio of medial (76%) to lateral (24%) meniscus tears [34].

Regarding isolated radial tears as a special entity of vertical meniscus tears, which are thought to mainly belong to the traumatic meniscus tears, no specific data are available for the incidence of these tears in stable knees in the literature. The rate of radial meniscus tears in patients undergoing knee arthroscopy was reported to range from 5 to 15% [2, 16, 35–38]. Unfortunately, the authors did not differentiate between stable and unstable or traumatic and degenerative tears [35, 37] *(Grade C).*

#### 6. What is the incidence of traumatic meniscus tears in unstable knees?

(a) Acute ACL + MCL tears: In knees with combined acute tears of the ACL and the medial collateral ligament (MCL), the incidence of lateral meniscus tears is higher than medial meniscus tears [39, 40], where a grade III MCL lesion appears to be protective compared to a grade II MCL lesion [40]. Numbers range from 32% for grade III MCL lesions and 71% for grade II MCL tears *(Grade C).*

(b) Acute ACL tears: In knees with acute tears of the ACL, the incidence of lateral meniscus tears is higher than medial meniscus tears (except ramp tears, please see below) [31, 34, 41–43]. The exact numbers vary significantly (16–82%) [44–47]. Approximately one-third to one-quarter of the patients do not have a meniscus tear [40, 41, 44, 48] *(Grade C).*

(c) Chronic ACL tears: In knees with chronic tears of the ACL, meniscus tears were reported in up to 96% of the patients [45-47]. The incidence of lateral meniscus tears is lower than that of medial meniscus tears [34, 41, 49, 50]. The rate of lateral meniscus tears is not substantially affected by the time after ACL tear and age. In contrast, the rate of medial meniscus tears increases over time and with increasing age [48, 49] *(Grade C).*

#### 7. What is the cause of pain in traumatic meniscus tears?

Traumatic meniscus tears themselves can cause knee pain [51, 52]. A traumatic meniscus tear can provoke pain by exerting a direct effect on the nociceptors of the meniscus and the synovial membrane [51, 53, 54] and through elevated concentrations of intra-articular cytokines [55] *(Grade C).*

### Diagnostics

#### 8. Are the clinical diagnostic tests accurate for assessing a meniscus tear of the knee?

A combination of diagnostic tests should be used to assess the meniscus, because this approach increases the accuracy [56–62] *(Grade C)*. Single tests only exhibit low to moderate diagnostic accuracy. A fair recommendation might be to use the McMurray joint line tenderness test because of its high sensitivity and specificity [56, 63–68] *(Grade A)*. The Ege and Thessaly tests have also a high sensitivity and specificity, but studies using these tests are scarce [57, 63] (Table 1) *(Grades A and C).*

**Table 1:** Sensitivity, specificity and accuracy of clinical tests to diagnose meniscal tears in different studies

Study	Type of study	Level of evidence	Number of studies	Number of patients	Diagnostic test	Medial/lateral meniscus	Sensitivity [%]	Specificity [%]	Accuracy [%]
Meserve et al. [198]	Systematic review	I	8	–	McMurray test	Both	52	97	N/A
			8	–	Joint line tenderness	Both	76	77	N/A
			3	–	Appley test	Both	22	88	N/A
Karachalios et al. [66]	Prospective cohort study	I	–	213 (157 M/56 F)	McMurray test	Medial	48	94	78
						Lateral	65	86	84
					Joint line tenderness	Medial	71	87	81
						Lateral	78	90	89
					Apley test	Medial	41	93	75
						Lateral	41	86	82
					Thessaly 5° of flexion	Medial	66	96	86
						Lateral	81	91	90
					Thessaly 20° of flexion	Medial	89	97	94
						Lateral	92	96	96
Eren [65]	Prospective Cohort study	III	–	104 (104 M)	Joint line tenderness	Medial	86	67	74
						Lateral	92	97	96
Jackson et al. [197]	Systematic review	III	4	–	McMurray test	Both	52	97	N/A
					Joint line tenderness	Both	76	29	N/A
Evans et al. [196]	Prospective cohort study	I	–	164	McMurray test	Both	16	98	N/A
Akseki et al. [63]	Prospective cohort study	II	–	150 (110 M/40F)	McMurray test	Medial	67	69	66
						Lateral	53	88	82
					Joint line tenderness	Medial	88	44	71
						Lateral	67	80	77
					Ege test	Medial	67	81	71
						Lateral	64	90	84
Konan et al. [67]	Prospective cohort study	III	–	109 (80M/29F)	McMurray Test	Medial	50	77	57
						Lateral	21	94	77
					Joint Line Tenderness	Medial	83	76	81
						Lateral	68	97	90
					Thessaly 5° of flexion	Medial	68	77	49
						Lateral	89	30	71
					Thessaly 20° of flexion	Medial	59	67	61
						Lateral	44	86	80
					McMurray + Joint Line	Medial	91	91	N/A
						Lateral	75	99	N/A
					Joint Line + Thessaly	Medial	93	92	N/A
						Lateral	78	99	N/A
Fowler and Lubliner [195]	Prospective cohort study	I	–	161 (106M/55F)	McMurray Test	Both	29	95	N/A
					Joint Line Tenderness	Both	85	30	N/A
					Apley Test	Both	16	80	N/A
Kurosaka et al. [68]	Prospective cohort study	III	–	156 (83M/73F)	McMurray Test	Both	37	77	45
					Joint Line Tenderness	Both	55	67	57
					Apley Test	Both	13	90	28
Gobbo et al. [56]	Prospective cross-sectional study	III	–	162 (117M/45F)	McMurray Test	Medial	65	58	61
						Lateral	62	49	52
					Apley Test	Medial	50	65	57
						Lateral	50	60	57
					Steinmann I Test	Medial	70	56	63
						Lateral	59	44	48
					Steinmann II Test	Medial	68	56	62
						Lateral	59	45	49
					All tests together	Medial	89	31	60
						Lateral	86	24	40
Corea et al. [194]	Prospective cohort study	II	–	93	McMurray Test	Medial	65	93	N/A
						Lateral	52	94	N/A
Solomon et al. [193]	Systematic review	III	4	–	McMurray	Both	53	59	N/A
					Joint Line Tenderness	Both	79	15	N/A

*M* male, *F* female

#### 9. Is an MRI systematically necessary in a knee with a suspected traumatic meniscus tear?

No consensus exists for this question. In addition to clinical experience, the systematic use of MRI also depends on the availability and legal issues in the different European countries. In general, MRI is a useful preoperative tool with a high accuracy for discriminating meniscus tears and other pathologies [37, 69–78]. However, if an arthroscopy is required, the usefulness of an MRI might be questioned [79–81]. It might help to improve planning of the surgery and inform the patient [82, 83].

The consensus group agrees that a musculoskeletal specialist should select the indication for an MRI *(Grade D).*

#### 10. Has a consensus been established for the assessment of meniscus healing?

Several different possibilities exist to assess meniscus healing. The most reliable technique to assess meniscus healing is arthroscopy [84–88]; however, it is still a subjective examination that depends on the surgeons’ skills. Magnetic resonance imaging (MRI) scans have mainly been used to evaluate meniscus healing, but signal changes persist for a long time and often do not correlate with clinical symptoms [86, 89–91]. Changes in MRI may occur even in asymptomatic knees. Thus, when the healing status is uncertain, magnetic resonance arthrography (MRA) might be a better choice than blank MRI [92–94]. Researchers have not determined whether direct (= intraarticular) or indirect (= intravenous) MRA should be used [94]. In the case of contraindications, arthro-computed tomography (CT) is a good alternative [91, 95], but it uses radiation. Both MRI and CT scans have the advantage that they are able to be assessed by different physicians as can clear arthroscopic pictures *(Grade B).*

#### 11. Factors affecting the success rate of repaired traumatic meniscus tears

##### 11.1 Does the location of a traumatic meniscus tear (zones 0–3) play a role in successful repair?

Yes, the location of a traumatic meniscus tear plays a role in the failure rate after repair. Repaired tears in Cooper zones 1 and 2 lead to excellent and good clinical mid-term results (from 64 to 91%) [96–100]. However, tears located in zone 1 have a statistically significantly better healing rate (from 87 to 91%) than tears located in zone 2 (from 59 to 79%) [84, 101, 102].

Furthermore, some studies have reported good clinical outcomes (from 75 to 87%) in selected patients with tears located in zone 3 [103–106] or have reported no correlation between the location of the tear and the results [107]. Thus, we concluded that the location of the tear in this zone should not be considered as an absolute contraindication for meniscus repair *(Grade C).*

##### 11.2 Does the location of the traumatic meniscus tear (anterior or posterior horn or pars intermedia) play a role in successful repair?

The anterior-to-posterior location of a traumatic meniscus tear does not appear to affect the surgical outcome [107]. However, the literature is very scarce *(Grade C)*.

##### 11.3 Does the length of a repaired longitudinal traumatic meniscus tear play a role in successful repair?

The literature is controversial regarding whether the length of a longitudinal meniscus tear affects the success of a repair [84, 86, 107–120]. Thus, the length of the meniscus tear should not be a contraindication for repair or partial meniscectomy *(Grade C).*

##### 11.4 Does the patient’s age affect the success of the meniscus repair?

The patient’s age does not appear to affect the failure rate of repairs of traumatic meniscus tears (available studies included patients with ages ranging from 9 to 58 years). However, the degeneration of the meniscus tissue in older patients should be considered [84, 85, 96, 99–103, 121, 122] *(Grade C)*.

##### 11.5 Does the patient’s BMI or weight affect the success of meniscus repair?

Although a higher BMI increases the likelihood of a degenerative meniscus lesion [123–125], patients with a higher BMI (up to 35) do not appear to have a higher risk for failure of meniscus repairs [126] *(Grade C)*.

##### 11.6 Does the patient’s level of activity affect the success of meniscus repair?

Controversial results were reported in the literature regarding the correlation between patients’ activity after meniscus repair and its success or failure [21, 96, 127–129]. Thus, no recommendation can be provide whether patients should go back to their preinjury activity level *(Grade C)*.

##### 11.7 Does lower limb alignment affect the success of repaired traumatic meniscus tears?

In contrast to the case for degenerative meniscus lesions, a clear relationship has not been observed between joint alignment and the recommended treatment for a traumatic meniscus tear. No particular studies have investigated the question of whether a traumatic meniscus tear in a varus or valgus knee should be treated differently compared to a straight knee *(Grade D)*.

#### 12. What is the fate of traumatic meniscus tears left in situ?

No results are available regarding the self-healing potential of isolated meniscus tears to our knowledge.

Meniscus tears left in situ at the time of ACL reconstruction have a low rate of reoperation (from 0 to 30%). Lateral meniscus tears appear to have a better prognosis in terms of secondary partial meniscectomy than medial meniscus tears left in situ (79–100% vs. 63–100%, respectively) [19, 24, 107, 114, 130–143].

Notably, most studies examined stable meniscus tears left in situ without treatment [24, 25, 114, 118, 135, 138, 140–144]. Nevertheless, Shelbourne and Heinrich included eight unstable meniscus tears in their study. None of the patients required a subsequent surgery [139]. One must also consider that some studies did not repair or partially resect the meniscus tear, but instead performed ‘biological’ treatments, such as rasping or needling.

Divergent results were identified in the literature regarding the correlation between tear length and failure rate of tears left in situ. Some studies reported a statistically significant higher rate of reoperations when the tear was longer than 10 mm [131, 135]. However, other studies did not observe a significant correlation [107, 140] or did not consider the length of the tear as a criterion [138, 142, 145] *(Grade C)*.

Thus, in general, small tears (≤ 10 mm) of the lateral meniscus can be left alone and do not require repair or partial meniscectomy. Tears of the medial meniscus should be repaired *(Grade D)*.

#### 13. What are the indications for the different treatment options for longitudinal traumatic meniscus tears in stable knees?

Preservation of the meniscus is the first-line option because the clinical and radiological long-term outcomes are worse after partial meniscectomy than meniscus repair [8, 9, 69, 146–149].

In general, traumatic meniscus tears are treated with repair, left in situ, or partial meniscectomy. Repair and left in situ are the most favorable treatment options, whereas the latter option is recommended for stable tears of the lateral meniscus during ACL reconstructions (see question 9) [8, 9, 24, 69, 84, 103, 106, 121, 132, 135, 138, 140, 143, 145–147, 149, 150]. Thus, repair is recommended for medial meniscus tears, unstable tears, such as bucket handle and double longitudinal tears, and isolated meniscus tears [84, 103, 106, 146].

To date, repair and left in situ repairs have not been compared directly, but the healing rates for tears of the lateral meniscus during ACL reconstruction appear to be comparable for all three meniscus zones (zones 3 to 1) *(Grade C)*.

Partial meniscectomy of traumatic meniscus tears should only be applied if the other two treatment options are not applicable, e.g., in complex tears, tears with a high degree of degeneration, flap tears or nonreducible bucket handle tears *(Grade D)*.

#### 14. What are the indications for the different treatment options for radial traumatic meniscus tears (except root tears) in stable knees?

(a) Complete radial tears may exert a detrimental effect on the knee, because they potentially represent an almost complete loss of meniscus function. In general, traumatic radial meniscus tears are treated with repair, left in situ or partial meniscectomy. Radial tears of zones 1 and 2 should be repaired to restore the integrity of the rim in patients with or without concomitant ACL reconstruction [69, 103, 106, 149, 151–153]. Only when the tear is technically not repairable or a retear of a failed repair occurs should partial meniscectomy be considered. Partial meniscectomy should not be the first-line treatment for tears of zone 1 and 2, because of its worse long-term outcome than repaired tears [8, 69, 149] *(Grade C)*.

(b) Nontreatment of the radial tear was also described as a treatment option for stable tears in all three zones (1–3) during concomitant ACL reconstruction [24, 69, 132, 135, 139, 145, 149]. Despite the good clinical results, the healing rates of repeated arthroscopic surgeries were very low, and these studies were only mid-term follow-up studies. Thus, this treatment approach is not recommended *(Grade D)*.

(c) In contrast, radial tears of zone 3 can be treated with a partial meniscectomy that preserves the peripheral wall. Some surgeons also prefer to repair the torn part of zones 1 and 2 and perform a partial resection of zone 3. However, no research studies compared this combined treatment with the repair of all three zones *(Grade D)*.

#### 15. Should posterolateral meniscus root tears be repaired?

Posterolateral meniscus root tears (PLMRT) are mainly traumatic injuries that frequently occur with ACL tears. These root tears should be repaired, particularly if the meniscofemoral ligament does not exist or is injured [84, 154]. The repair of PLMRT decreases meniscus extrusion (in the sagittal plane) and the risk of OA compared to untreated tears in patients with concomitant ACL reconstruction. However, no differences were observed in Lysholm scores and objective IKDC grading between treated and untreated PLMRT [155, 156] *(Grade C)*.

#### 16. Should a posteromedial meniscus root tear be repaired?

Medial meniscus roots tears may be traumatic; however, they generally present a degenerative nature. Root repair is typically recommended because it results in a better clinical outcome than conservative treatment and partial meniscectomy [157, 158] and may also reduce the risk of progression of OA. The degree of OA plays an important role in determining the outcome: the higher the degree of OA, the less favorable the results *(Grade C)*.

#### 17. What is the optimal timing of a successful repair of stable and unstable meniscus tears?

A repair that is completed as early as possible appears to produce a better clinical outcome, including a decreased failure rate (Table 2). In general, acutely repaired meniscus tears achieve superior results compared to chronically repaired tears (Table 2). However, repaired chronic meniscus tears achieve good to excellent results and thus should be repaired, when indicated, instead of partially resected (Table 2) *(Grade C)*.

**Table 2:** Success rates of meniscus repairs depending on time of repair after tear

Study	Type of study	Level of evidence	Number of patients	Treatment	Time cutoff	Acute/chronic	Success rate [%]
Steenbrugge et al. (2002)	Prospective study	IV	13(7 M/6F)	Meniscal repair	2 weeks	Acute	100
						Chronic	80
Stone et al. [100]	Prospective study	IV	31	Meniscal repair	2 weeks	Acute	100
						Chronic	64
Cannon and Vittori [111]	Prospective study	IV	90	Meniscal repair	8 weeks	Acute	88
						Chronic	79
Buseck and Noyes	Prospective study	IV	66(21 M/45F)	Meniscal repair	8 weeks	Acute	97
						Chronic	90
Barrett et al. [121]	Prospective study	IV	37(26 M/11F)	Meniscal repair	8 weeks	Acute	89
						Chronic	78
Eggli et al. (1995)	Prospective study	IV	54	Meniscal repair	8 weeks	Acute	90
						Chronic	71
Noyes et al. (2000)	Prospective study	IV	27	Meniscal repair	10 weeks	Acute	90
						Chronic	85
Venkatachalam et al. (2001)	Prospective study	IV	59(38 M/21F)	Meniscal repair	3 months	Acute	92
						Chronic	58

#### 18. What is the best method to treat an acute, nonreducible (= locked) bucket handle meniscus tear in combination with an ACL tear in the office or emergency room?

(a) Chronic ACL tear: In a noninflammatory knee with an ACL injury that occurred several weeks or more ago and an acute, nonreducible bucket handle meniscus tear, the preferred treatment is the prompt repair of the meniscus and the simultaneous reconstruction of the ACL [159, 160]. The group does not recommend a two-stage treatment with an initial repair of the meniscus tear and secondary reconstruction of the ACL, as proposed by other researchers [105, 161] *(Grade C)*.

(b) Acute ACL tear: Similarly, in an acutely injured knee with an ACL tear and a nonreducible bucket handle meniscus tear, the preferred treatment is the prompt repair of the meniscus and the possible reconstruction of the ACL in the same procedure. One study recommended a concomitant ACL reconstruction and meniscus repair if it was able to be completed within approximately 60 h after the injury [162]. An increase in the arthrofibrosis rate was not reported when patients received ACL reconstruction within the first 60 h compared to delayed surgery *(Grade C)*.

(c) Subacute ACL tear (inflamed knee): Controversy exists regarding the procedure for treating subacute cases with a ACL tear that occurred several days prior to surgery and a clinically nonreducible bucket handle meniscus tear in an inflamed knee (= knee irritation, effusion, swelling) [163]. The first treatment option is the repair of the bucket handle meniscus tear and the reconstruction of the ACL (one-stage surgery), which poses a risk of arthrofibrosis but protects the repaired meniscus [164]. Alternatively, a two-stage approach can be chosen in which the meniscus tear is repaired first, followed by the reconstruction of the ACL. ACL reconstruction can be performed after full ROM is attained and the knee is no longer inflamed [105]. This approach would also offer the patient time to heal between the two surgeries, to prepare for the ACL reconstruction, and to assess the meniscus healing during the second surgery [161]. The ACL reconstruction should not be delayed too long because the risk of meniscus (re-) tear increases by 1% with each 1-month interval from injury to surgery [165, 166]. Because repaired menisci do not perform well in ACL-deficient knees, and a new meniscus tear may develop in these knees, patients may wear a brace between the surgeries to potentially protect the menisci [165–167]. One-stage and two-stage approaches were not compared in the literature in subacute patients with inflamed knees. These approaches were only compared in chronic patients and produced controversial results [105, 159]. Therefore, the prompt repair of the meniscus is recommended, but no recommendation is proposed regarding whether the ACL should be reconstructed simultaneously or after inflammation is resolved and full range of motion (ROM) is attained *(Grade D)*.

#### 19. What are the indications for partial meniscus substitution?

(a) The implantation of partial meniscus replacements (PMR) of the medial meniscus during the first partial (subtotal) resection is a topic of discussion, as the only level-1 study showed no benefit of PMR compared to isolated partial meniscectomy [168, 169]. Thus, the implantation of a PMR within the first surgery for partial meniscectomy is not generally recommended. Partial meniscus replacement may be considered for patients with failed meniscus surgeries and meniscus-related complaints [168, 170–172]. *(Grade A)*.

(b) Results for lateral and medial PMR are similar, according to one study [173] *(Grade C)*.

(c) According to one study, the implantation of a PMR (collagen meniscus implant = CMI) in combination with ACL reconstruction produced a superior outcome in the first surgery compared to a partial meniscectomy alone [174]. However, these results should be interpreted with caution, because it was retrospective study that included a heterogeneous group of patients *(Grade C)*.

#### 20. Are biological techniques useful to enhance meniscus healing?

A. Does RASPING of a meniscus tear and/or the surrounding synovial membrane enhance meniscus healing?

Although rasping is an easy, cheap and quickly performed technique, researchers have not clearly determined whether rasping is appropriate to enhance meniscus healing. Studies evaluating patients with isolated meniscus tears are lacking. Only a few studies are available that included patients with concomitant ACL rupture. These studies reported controversial results regarding meniscus rasping [19, 175]. Concomitant ACL reconstruction positively affects meniscus healing; thus, it is a severe confounder in the aforementioned studies [111, 176–178] *(Grade C).*

B. Does NEEDLING of the meniscus tear enhance the healing of a meniscus tear?

Studies on the needling of isolated full-thickness traumatic meniscus tears are lacking [179, 180]. Thus, no evidence reported to date supports the use of this treatment, and surgeons should apply it with caution. In the case of using needling of a meniscus to enhance healing, the surgeon must carefully consider the potential of the needle used in this technique to damage the meniscus [181] *(Grade C).*

C. Does OPENING THE MEDULLARY CAVITY enhance meniscus healing?

Clinical studies of the ability of this method to enhance meniscus healing in patients with isolated meniscus repairs are lacking. Thus, no statement can be made. *Grade:* n/a.

D. Does the local application of FIBRIN GLUE enhance meniscus healing?

Studies evaluating patients with isolated (without concomitant ACL reconstruction) meniscus repair performed with fibrin glue are lacking. Only two studies published by the same study group have evaluated the effect of locally applied fibrin glue into repaired meniscus tears [182, 183]. Nevertheless, both studies included several patients with concomitant ACL reconstruction. Concomitant ACL reconstruction positively affects meniscus healing [111, 176–178]. Thus, the use of locally applied fibrin glue to enhance meniscus healing may be considered, but is not currently recommended *(Grade C).*

E. Does the local application of a FIBRIN CLOT enhance meniscus healing?

In general, the use of an isolated fibrin clot is not recommended for the treatment of traumatic full-thickness meniscus tears due to a lack of studies. For radial tears, weak evidence of a positive effect of fibrin clots that have been locally applied into meniscus tears exists, based on promising results from a small case series [152, 184] *(Grade C).*

F. Does the local application of PLATELET-RICH PLASMA (PRP) enhance meniscus healing?

The additional use of PRP during the repair of traumatic meniscus tears is not recommended. The only available study did not show an improvement when using PRP compared to isolated meniscus repairs [185]. (*Grade C).*

G. Does the LOCAL APPLICATION OF CELLS enhance the healing of a meniscus tear?

The local application of cells to enhance meniscus healing is not recommended, because no published studies have evaluated the isolated effects of cells on meniscus healing (with or without sutures) *Grade: n/a.*

## Discussion

The main message of this consensus is that the preservation of the meniscus should be the first choice of treatment for traumatic meniscus tears, because of its excellent outcome in terms of the return to a high level of activity and OA prevention, as evidenced by the agreement between the literature and expert opinion. The preservation of the meniscus is of utmost importance because menisci play important roles in load distribution, joint stabilization, neuromuscular function, lubrication and cartilage nutrition [186–188]. Approximately 30% of meniscus tears are potentially repairable, and only up to 10% are currently repaired [13]. The main reasons for this gap appear to be faster recovery after meniscectomy, lower costs of the surgical procedure and the risk of revision surgery [189]. Several factors are considered responsible for higher failure rates, such as an older age of patients, high BMI, chronicity of tears, tear length or tear location in Cooper zone 3. In this consensus, none of these factors were contraindications for meniscus repair. In particular, repair should be performed for longitudinal tears with a length greater than 10 mm, including bucket handle tears, radial tears of Cooper zones 1 and 2, and root tears. This recommendation also includes late repairs, if technically reasonable, because healing rates are still very good, even for late repairs. These tears frequently cause a complete loss of meniscus function. Early repair may produce a better outcome than delayed repair. Thus, although patients rapidly return to work and sports after partial meniscectomy, the costs of initial surgery are lower and the failure rate is lower than meniscus repairs, the significant inferior long-term outcomes of patients with partially resected menisci compared to repairs in terms of OA and the return to a high activity level highlight the value of repairing meniscus tears [7, 9, 190].

Meniscus tears rarely occur in isolation and are often associated with anterior cruciate ligament (ACL) injuries. Ideally, both pathologies are addressed during the same surgery. Isolated meniscus repairs in unstable knees, such as an ACL-deficient knee, should be avoided because of their high failure rate. Additionally, meniscus repairs performed with concomitant ACL reconstruction show a higher healing rate. The explanations for this higher healing rate might be attributed to bone marrow-derived stem cells originating from ACL tunnel drilling, a more conservative rehabilitation protocol after ACL reconstruction, or the surgically induced formation of a hematoma that releases specific growth factors and stem cells. However, the concomitant treatment of meniscus and ACL tears might be a challenging decision such as during the first few days after knee injury in a patient whose knee has acute inflammation with a large effusion, a range of motion deficit and bucket handle tears, which was not reducible conservatively and thus requires surgery. In general, in the case of inflammation in the knee, knee surgery would be delayed until symptoms have disappeared. However, a bucket handle meniscus tear should be reduced and repaired as early as possible to result in the highest possibility of a successful repair and to avoid the unnecessarily prolonged suffering of the patient. Under these specific circumstances, instant ACL reconstruction may be performed concomitantly with meniscus repair, even after considering the risk of arthrofibrosis. Another option would be to reduce and repair the meniscus and to stabilize the knee with an orthosis until the inflammatory phase, including effusion, resolves and ROM is acceptable, allowing a safer ACL reconstruction.

An interesting topic that has attracted increasing attention over the last few years is the use of so-called biologicals or biological techniques to improve meniscus healing. These techniques are mainly used concomitantly with meniscus repair and include the local application of platelet-rich plasma, stem cells, blood clots, and fibrin glue. Furthermore, needling of the meniscus tissue around the tear, rasping of the meniscus tear and surrounding synovial membrane, as well as opening of the medullary cavity are also biological techniques. Interestingly, during the period of establishing the consensus, none of these techniques had been confirmed to enhance meniscus healing in humans. However, a recent study showed a positive effect of the latter technique on meniscus healing. In this study, similar healing rates were observed in patients undergoing meniscus repairs with concomitant ACL reconstruction and isolated meniscus repairs with opening of the medullary cavity [191].

In addition to the patient’s history, a clinical examination is required to diagnose traumatic meniscus tears. The McMurray test shows the highest sensitivity and specificity. Nevertheless, it still has a limited accuracy. To improve the sensitivity and specificity, a special score has been introduced to improve the sensitivity and specificity that includes the following five criteria: (1) the history of locking and catching, (2) pain upon hyperextension and (3) hyperflexion, (4) pain when palpating the joint line and (5) a positive McMurray test. If all these criteria are met, the predictive value is 92%, and the predictive value of four and three positive criteria is 82% and 77%, respectively [192]. In addition to a clinical examination, MRI plays an important role in diagnosing meniscus tears. Thus, MRI should be available prior to surgery mainly for two reasons. It will help identify the type of meniscus tear, which may significantly affect the choice of the type of surgery, and it may be useful to diagnose associated pathologies. The most commonly associated pathologies are ACL injuries, which are sometimes difficult to diagnose, e.g., in patients with bucket handle tears. Interestingly, a 3 Tesla MRI does not appear to significantly improve the sensitivity and specificity in diagnosing meniscus tears compared to a 1.5 Tesla MRI if a good knee coil is used [78].

This consensus has some limitations. The scientific level of evidence of the consensus is only as good as the literature. The analysis of the literature revealed a limited number of studies with a high level of evidence available. The validation of all questions and answers by the rating and peer review groups provides important input based on the expert opinions of orthopedic surgeons with a special interest in this field. Therefore, a consensus should not be considered as a guideline for the treatment of traumatic meniscus tears, but it does provide the best recommendation possible for the treatment of traumatic meniscus tears based on the current scientific evidence and clinical expertise. Since the knowledge on this topic will continue to evolve with further studies, deeper insights could be obtained in the future.

## Conclusions

The consensus was generated to present the best possible recommendations for the treatment of traumatic meniscus tears and provides some groundwork for a clinical decision-making process regarding the treatment of meniscus tears. Preservation of the meniscus should be the first line of treatment when possible because the clinical and radiological long-term outcomes are worse after partial meniscectomy than after meniscus preservation. The consensus clearly states that numerous meniscus tears that were considered irreparable should be repaired, e.g., older tears, tears in obese patients, long tears, etc.
